# Medical Society of the County of Erie

**Published:** 1896-07

**Authors:** Franklin C. Gram

**Affiliations:** Secretary


### MEDICAL SOCIETY OF THE COUNTY OF ERIE.

Reported by FRANKLIN C. GRAM, M. D., Secretary.

THE semi-annual meeting of the Medical Society of the County of Erie was held Tuesday, June 9, 1896, in the rooms of the Buffalo Academy of Medicine, 617 Main street. The president, Dr. J. G. THOMPSON, called the meeting to order at 10.15 o'clock A. M. The minutes of the annual and of the special meetings were read and approved.

Dr. T. M. JOHNSON, of the committee on membership, reported in favor of admitting the following-named applicants to membership: Drs. H. C. Rooth, Martha F. Caul, Wellington G. Grove, Richard H. Satterlee, J. Grafton Jones, Simon Clug, John R. McCarthy, Chester T. Stewart, F. E. Luke, Henry Osthues and J. Henry Dowd. They were elected by unanimous vote.

Applications for membership were received from Drs. Jane N. Frear, Frederick W. Hayes, Edward E. Koehler, Earl P. Lothrop, E. T. Rulison, Albert E. Woehnert, Marion Marsh, Cora Billings Lattin and Henry W. Lattin. Referred to the committee on membership.

Dr. M. D. MANN, of the committee selected at the special meeting to attend a hearing before the state legislature in the interests of medical legislation, made a verbal report. He also presented a bill for $9.12, one-half of his expenses to Albany, the other half being paid by the University of Buffalo, which he also represented. Dr. Mann was tendered a vote of thanks and his bill was ordered paid.

Dr. Ernest Wende, of the committee appointed to consider the propriety and investigate the feasibility of establishing a professional home for the several medical societies of this county and city, reported that from a preliminary canvass made among physicians and interested citizens the committee considered the project entirely feasible. He, therefore, reported progress and asked for a continuance of the committee, which was granted.

Dr. J. B. Coakley, chairman of the Board of Censors, submitted the following report:

*To the Medical Society of the County of Erie:*

The Board of Censors respectfully presents its semi-annual report as follows:

At the time of our last report the case of D. R. Burton, of 1902 Niagara street, Buffalo, N. Y., a so-called faith healer, was laid before the society. We secured what we supposed would be sufficient evidence to procure an indictment under chap. 398 of the laws of 1895 and furnished the same to the district attorney, who brought the matter before the grand jury. After deliberating upon the case they failed to report an indictment.

In the minds of this board Burton is clearly violating the spirit, if not the letter, of this law. If the law has not been framed explicitly enough to cover such cases as this we feel that the fact should be made to appear clearly by an actual test, and the law then amended so as to secure successfully the object for which it was designed.

We feel that if we were authorised by this society to employ counsel to assist in working up this case more thoroughly and then in laying it before the district attorney and grand jury an indictment would probably be secured and a good test case made.

This board, equipped with proper legal assistance and working for the honor of the profession and the suppression of quackery, would probably be able to secure more decisive results than would follow if such cases were turned over for investigation entirely to the district attorney's office, already overcrowded with the multitude of matters and having no particular interest in the result beyond the proper performance of duty.

As previously reported, the district attorney has expressed to this board his distrust of the strength of the language and the definiteness of this law under which we seek to secure the conviction of faith-heal ers and the like. The defectiveness of this law, if such there be, can only be proved by a test case earnestly fought out in the courts.

We, therefore, recommend that the Board of Censors be authorised to employ counsel for the purpose of working up this Burton case properly, or such other stronger one as may be found, and so of testing the strength of this recent law.

Under this law a first offense may be punished by a fine of not more than $250 or by imprisonment for six months, and a subsequent offense by a fine of not more than $500 or imprisonment for a year, or by both fine and imprisonment. We quote further from the same law as follows: "When any prosecution under this article is made on complaint of any incorporated medical society of the state or any county, medical society the fine when collected shall be paid to the society making the complaint, and any excess of the amount of fines so paid over the expense incurred by the said society in enforcing the medical laws of this state shall be paid at the end of the year to the county treasurer."

One other case was called to the attention of this board in April last by Dr. H. R. Hopkins—namely, that of Dr. Ballentine, under whose name an advertising business is being conducted at Room 4, Mooney building, Buffalo. We have thoroughly investigated this matter also and find that Dr. Ballentine is not personally in this city, but that there are three physicians practising in that office and representing Dr. Ballentine, all of whom are regularly licensed and registered, viz.: Dr. W. A. Crandall, registered April 6, 1896; Dr. T. V. Koons, registered April 10, 1896, both of whom are of the homeopathic school, and Dr. Lefferts M. Powell, registered March 31, 1896, of the eclectic school. While violating the ethics of the profession manifestly, and while it seems that their plan of action is merely to force druggists to keep Ballentine's remedies in stock, and though it is certainly reprehensible to physicians to lend themselves to such a scheme, we can find no violation on their part of the law of this state.

Respectfully submitted.

J. B. COAKLEY.
FRANCIS METCALFE.
MARCELL HARTWIG.
HENRY LAPP.

It was stated that there were some fifteen or twenty alleged physicians practising in Buffalo in violation of the law. Dr. Coakley said that those who knew of any such cases should communicate their information to the board in writing and they would be promptly investigated.

On motion the report was received and the recommendations therein were adopted.

Dr. J. J. Walsh stated that the committee appointed to consider the county hospital question had never held a meeting, and on his motion the committee was continued until the annual meeting.

The President stated that he had been requested to appoint five delegates to represent this society in the Buffalo School Association, and had appointed as such Drs. H. R. Hopkins, William Warren Potter, Wm. C. Krauss, F. C. Gram and J. C. Green.

On motion the president's action was approved.

The SECRETARY stated that the papers read at the annual meeting were nearly all published and would soon be issued in pamphlet form to the members. He also read an acknowledgment from the State Board of Health of the resolutions endorsed at the annual meeting in connection with the question of tuberculosis among the Indians of this state.

Dr. NELSON G. RICHMOND, of Fredonia, N. Y., read a paper on Acute epiphysitis, which was discussed by Drs. Mynter, Hayd and Cronyn.

Dr. R. H. SATTERLEE'S paper on Refraction without mydriatics was discussed by Drs. Hubbell, Howe and Starr.

Dr. A. W. HURD, superintendent of the Buffalo State Hospital, read a paper on Early treatment of mental diseases, in which he also called attention to the new lunacy law. Drs. Krauss, Putnam and Crego discussed the subject.

Forced breathing and the pneumatic cabinet was the subject of a brief paper by Dr. J. H. Pryor. The discussion of this was led by Dr. Rochester.

Shortly after 1 o'clock P. M. the society adjourned.

